# Endometriosis and Ovarian Cancer: A Systematic Review

**DOI:** 10.5402/2011/140310

**Published:** 2011-07-15

**Authors:** Ahmad Sayasneh, Dimitris Tsivos, Robin Crawford

**Affiliations:** Department of Obstetrics & Gynaecology, The Rosie Hospital, Cambridge University Hospital NHS Trust, Hills Road, Cambridge CB2 2QQ, UK

## Abstract

*Introduction.* Endometriosis is one of the most common benign disorders which affects 10–15% of all women in reproductive age. The association between endometriosis and ovarian cancer has been frequently described in the medical literature. *Purpose.* To evaluate the literature for evidence of a correlation between endometriosis and ovarian cancer. *Method.* the English language literature (online MEDLINE and EMBASE database) was searched using the keywords endometriosis combined with cancer, tumour, tumor, carcinoma, or adenocarcinoma. All abstracts between January 1985 and August 2010 were reviewed. Full relevant articles were critically assessed. Reference lists of included studies were checked. *Results.* Seven out of the eight studies, included in our review, have shown an increased risk of ovarian cancer. However, the effect size is modest (OR, RR, and SIR) ranging between 1.32 and 1.9 (95% CI). A causative relationship between the two incidences cannot be confirmed. There is increasing evidence on the role of genetic mutations in ovarian clear-cell and endometrioid carcinoma developing from endometriosis. *Conclusion.* More evidence is needed before suggesting any change in the current management of endometriosis.

## 1. Introduction

Endometriosis is one of the most common gynaecological disorders. It affects 10–15% of all women in the reproductive years [1]. The incidence is 40–60% in women with dysmenorrhoea and 20–30% in those with subfertility [1]. Although endometriosis is recognised as a benign disease, its association with ovarian cancer has been frequently described in the medical literature since 1925. In that year, Sampson established the first histopathological criteria, which are still in use, to identify malignant tumours rising from endometriosis: (1) clear evidence of endometriosis close to the tumour, (2) the carcinoma must be seen to arise in endometriosis, and not to be invading it from other sources, and (3) presence of tissue resembling endometrial stroma surrounding characteristic glands [2]. Later in 1953, Scott has added a fourth criterion which is the demonstration of a histology-proven transition from benign endometriosis to cancer [3]. The application of all these four criteria has rarely been fulfilled in the literature, which supports the idea that the malignant transformation of endometriosis is a rare event [4]. Yet, their stringent use may lead to underestimate the real frequency of this phenomenon [4]. 

The aim of this paper is to systematically review the literature evidence of a correlation between endometriosis and ovarian cancer.

## 2. Methods

A protocol-driven systematic review was conducted in accordance with the Centre for Reviews and Dissemination (CRD) guidance. [5] The English language literature (online MEDLINE and EMBASE database) was searched using the keywords: endometriosis combined with cancer, tumour, tumor, carcinoma, or adenocarcinoma. All abstracts between January 1985 and August 2010 were reviewed and full articles of relevant publications in English language were retrieved. A further systematic analysis of the publications included in the reference lists was performed. All up-to-date reviews on the same topic in the literature were assessed according to Glasgow appraisal tool [6]. Case reports and case series studies were excluded from our review.

## 3. Results

Seven reviews were found in the literature [4, 7–12], which have addressed the association between endometriosis and ovarian cancer. A summarizing table of the reviews' findings was modified from the Glasgow appraisal tool (Table 1) [6]. 

**Figure 1:**
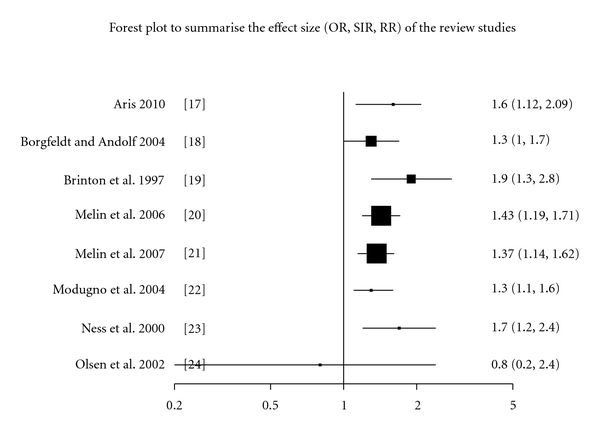
A Forest pilot summarises the eight studies' effect size. Effect size was measured in odd ratio (OR), standardized incidence ratio (SIR), or relative risk (RR). The 95% confidence interval is represented by the horizontal line, and the dimensions of the boxes are proportional to the sample size.

In our review, 11 studies were identified, which addressed the association between endometriosis and ovarian cancer [14–24]. To remove the selection bias, studies on the *subfertility* patients with endometriosis [14, 15] and studies on *endometriomas* instead of endometriosis [16] were excluded. Eight studies were included in the list [17–24]. Seven out of the eight studies have shown an increased risk of ovarian cancer. However, the effect size is modest (odd ratio OR, relative risk RR, and standardized incidence ratio SIR), ranging between 1.32 and 1.9 (95% CI) Figure 1.

In epidemiological terms, when the RR is less than 2, a careful assessment of the confounding factors must be conducted before any conclusion of causality can be made [25, 26]. The previous epidemiologic definition was considered when we selected the studies.  Table 2 summarises the types, sample size, followup time, confounding factors, and limitation of each one of the eight studies included in our review.

## 4. Discussion

Despite the studies presented, the risk of ovarian cancer among patients with endometriosis has always been *contentious*. Endometriosis is usually confirmed by laparoscopic or histological examination, and many patients are unaware of having the disorder. Therefore, identifying endometriosis as a preceding factor of ovarian cancer is not possible in many cases. On the other hand, the natural history of ovarian cancer is not well understood and the origin of the precursor cell, especially for endometrioid and clear-cell variants, is questionable. For these reasons, all studies in the literature would struggle to establish a causal relationship between the two entities. In this updated review we have tried to assess the epidemiologic evidence in the literature and to discuss our findings in view of the other genetic, immunological, and biological relevant studies. 

In our review we have found another group of eight epidemiologic studies that correlated endometriosis with endometrioid and/or clear-cell ovarian carcinoma as a specific histological subtype [27–34]. They all reported a specific link between endometriosis and endometrioid and/or clear-cell ovarian carcinoma, with an OR ranging between 3.7 and 35.4 (CI 95%). We have excluded these case series studies from our meta-analysis as there were not case-control or cohort studies. 

In a previous review, Somigliana et al. included two other studies which assessed the risk of endometriosis on ovarian cancer in infertile patients [4]. In the first study, infertile patients with endometriosis had the highest risk with an SIR of 2.5 (95% CI, 1.3–4.2) compared to the general population and an SIR of 4.2 (95% CI, 2.0–7.7) for the group with primary infertility [15]. In the second study, endometriosis and unknown cause of infertility resulted in an independently associated elevation in ovarian cancer risk after adjustment for standard confounding factors (odds ratios (OR) 1.7 (95% CI, 1.1–2.7) and 1.2 (95% CI, 1.0–1.4), resp.,) [14]. As infertility involves multiple confounding factors, and to remove the selection bias in our review, these two studies were excluded from our review.

Additionally, we have excluded another study conducted by Kobayashi et al.'s in Japan. They documented only endometriomas and evaluated the risk of ovarian cancer based on varying time periods from time of diagnosis of endometrioma [16]. Kobayashi et al. study did not account for patients with extraovarian endometriosis and only approximately one-third of these patients had surgically confirmed endometriomas, with the remaining diagnoses made based on ultrasonographic findings and physical exam only [16]. During followup of up to 17 years, 46 incidental ovarian cancers were identified, translating into a standardized incidence ratio of 8.95. This risk increased with age, with an incidence ratio of 13.2 in women over age 50 [16]. 

Many genetic, biological, and immunological studies have tried to address the causal relationship between endometriosis and ovarian cancer. Different types of genomic instability and mutations have been shown to occur in endometriosis and ovarian cancer [35, 36]. Moreover, microsatellite analysis has demonstrated that loss of heterozygosity on p16 (Ink4), GALT (galactose-1-phosphate uridylyltransferase) and p53, as well as on APOA2 (apolipoprotein A), a region frequently lost in ovarian cancer, occurs in endometriosis [37]. Another study by Baxter et al. has found the GSTM1 (glutathione S-transferase M1) null allele not to be an endometriosis susceptibility allele [38]. However, it may predispose endometriotic lesions to malignant transformation to endometrioid and clear-cell ovarian cancer [38]. Overexpression of p53, oncogenic K-ras Pten deletion, and loss of heterozygosity may also be involved in the endometriosis transformation to ovarian cancer [39–41]. 

A vital factor in the development of both endometriosis and malignancy is considered to be angiogenesis. In a study by Hayrabedyan et al., the expression of several angiogenic factors (interleukin-1 alpha (IL-1 alpha), Fibroblast growth factor FGF-1, and S100A13) and a common pan-ovarian carcinoma antigen were investigated, in several cases of adenomyosis and ovarian endometriosis [42]. They have shown that the common ovarian carcinoma marker, as well as these angiogenic factors, was expressed in most of the studied cases, implying possible common pathological mechanisms shared between endometriosis and malignancy [42]. In another study, Chou et al. illustrated that the cyclooxygenase-2 (COX-2) overexpression rate was higher in ovarian carcinoma associated with endometriosis than in isolated ovarian carcinoma (27.8% versus 5.6%, *P* = 0.083) [43]. They suggested that COX-2 over-expression may be a result of the malignant transformation of endometriosis to endometrioid type ovarian cancer or may represent an interaction between the two cellular components [43]. By contrast, Keita et al. suggested alteration in the expression of interleukin-1 receptor antagonist IL-1RA, a key protector against tumorigenic effects of IL-1, as a possible link between the endometrium, endometriosis, and endometrioid ovarian cancer [44].

Recently, Wiegand et al. published new data implicating ARID1A (AT-rich interactive domain-containing protein 1A) as a tumor-suppressor gene frequently disrupted in ovarian clear-cell and endometrioid carcinomas [45]. They have found ARID1A mutations in 55 of 119 ovarian clear-cell carcinomas (46%), 10 of 33 endometrioid carcinomas (30%), and none of the 76 high-grade serous ovarian carcinomas [45]. They demonstrated that the loss of the BAF250a protein was correlated strongly with the ovarian clear-cell carcinoma and endometrioid carcinoma subtypes and the presence of ARID1A mutations [45]. By comparing ovarian clear-cell carcinomas to their contiguous atypical endometriotic lesions in two patients, they have shown that the same mutations may be present in the putative precursor lesions and in the tumors. In contrast, the distant endometriotic lesions do not have ARID1A mutations [45].

It seems, from the previous discussion, that there is insufficient evidence to suggest a specific gene mutation or a specific biological pathway that predisposes endometriosis patients to ovarian cancer. There is good evidence, however, to demonstrate the potential transformation from endometriosis to ovarian endometriosis cell and clear-cell carcinoma. The association between the two entities with an effect size of 1.32–1.9 may be due to sharing similar risk factors, rather than a causal relation.

## 5. Conclusion

There is increased risk of ovarian cancers, specifically endometrioid and clear-cell carcinoma, in women with endometriosis. The estimated effect size, however, is modest varying between 1.32 and 1.9. A causative relationship between the two incidences cannot be confirmed. However, there is increasing evidence on the role of genetic mutations in ovarian clear-cell and endometrioid carcinoma developing from endometriosis. There are few gene mutations involved, and yet more evidence is needed before generalising any mutation screening test or changing the treatment of endometriosis to include radical excision in case of a positive genetic mutation.

## Figures and Tables

**Table 1:** A summery of the reviews' findings.

Review	Language of literature searched	Type of studies included	Quality assessment tool used in the review	Overall results	Application of results
Ness 2003 [7]	English	In vitro, animal, clinical, and epidemiologic studies	Not specified	Consistent with the association between endometriosis and ovarian cancer.	Possible chemoprevention for women with endometriosis.
Somigliana et al. 2006 [4]	English	Observational, cohort, and case-control	Studies have been critically analysed.	Increased risk of ovarian cancers: effect size: 1.3–1.9.	Modifications of the standard treatment options for the disease are not justifiable.
Vigano et al. 2007 [8]	English	Observational, cohort, and case-control epidemiologic, biological, and genetic studies	Nineriteria, by Austin Bradford Hill [13]	The criterion of strength has not been fulfilled. There were insufficient data for four criteria, and four criteria were fulfilled.	The low magnitude of the risk observed is consistent with the view that ectopic endometrium undergoes malignant transformation with a frequency similar to its eutopic counterpart.
Nezhat et al. 2008 [9]	English	Observational, cohort, and case-control epidemiologic, histopathological, and molecular studies	Not specified	Histological transition from benign endometriosis to ovarian malignancy.	The malignant potential of endometriosis holds serious implications for management.
Baldi et al. 2008 [10]	English	Not specified	Not specified	Further epidemiological and genetic studies are required.	Appropriate physical screening and imaging testing are recommended.
Vlahos et al. 2010 [11]	No search criteria specified	No search criteria specified	Not specified	Endometriosis is associated with specific types of ovarian cancer (endometrioid and clear cell).	More studies are needed to establish the risk factors that may lead to malignant transformation.
Kobayashi 2010 [12]	English	Studies on screening, epidemiology, clinical diagnosis, natural history, preclinical and clinical trials, and promising molecular targets on epithelial ovarian cancer (EOC).	Not specified	Ovarian endometrioma could be viewed as a neoplastic process.	Understanding the mechanisms of endometriosis development and elucidating its pathogenesis and pathophysiology are intrinsic to prevention.

**Table 2:** Summary the types, sample size, followup time, confounding factors, and limitation of each one of the eight studies included in our review.

Name of study	Type	Mean of followup (years)	Size of endometriosis cohort	Ovarian cancer cases identified in the cohort	Confounding factors considered	Main limitations
Aris 2010, Canada [17]	Retrospective cross-sectional	9	2521	41	Age, pregnancies, family history, race, oral contraceptive, tubal ligation, hysterectomy and breastfeeding	Retrospective collection of data using a coded computerised system. Selection bias
Borgfeldt and Andolf 2004, Sweden. [18]	Case-control	10	28,163	81	Age and parity	Use of cohort of women discharged from hospital with a diagnosis of endometriosis. This may lead to including women with moderate and severe endometriosis (hospital stay patients) without minimal and mild cases. This may overestimate the risk ratio. Selection bias.
Brinton et al. 1997, Sweden. [19]	Retrospective cohort study	11.4	20,686	29	Age and length of history of endometriosis	
Melin et al. 2006, Sweden. [20]	Retrospective cohort study	12.7	64,492	122	Age and type of surgery performed.	
Melin et al. 2007, Sweden. [21]	Retrospective cohort study	13.4	63 630	134	Age, parity, and type of surgery performed	
Modugno et al. 2004, USA. [22]	Case-control			177	Age, parity, oral contraceptive use, tubal ligation, family history of ovarian cancer, and study site, gynaecological surgical procedures	Recall and selection bias, as the authors pooled data on the history of endometriosis reported by patients.
Ness et al. 2000. USA. [23]	Case-control			66	Age, history of ovarian cancer, parity, breastfeeding, type and length of each contraceptive, tubal ligation, hysterectomy, ovarian operations.	Data was collected by case interviews. Recall bias. Low participation rates among cases and controls.
Olsen et al. 2002, USA [24]	Prospective cohort study.	13	1,392	3	Age, education, marital status, alcohol intake, physical activity, smoking, parity, oral contraceptive use, HRT, history of hysterectomy or dilatation and curettage, BMI and waist to hip ratio.	Reliance on self-reports of endometriosis in this cohort (a questionnaire). Recall bias. The number of women who developed ovarian cancer is quite limited.
